# Evaluation of various filter paper and reagent systems for the preservation of Newcastle disease virus RNA samples

**DOI:** 10.2478/jvetres-2025-0030

**Published:** 2025-06-09

**Authors:** Bajes Amjed Al Qaisieh, Mustafa Mohammed-Khair Ababneh, Mohammad Borhan F. Al-Zghoul, Daoud Abed Alnaser Alghizzawi, Hebah Alaeddin Aboomer

**Affiliations:** 1 Department of Basic Medical Veterinary Sciences, Faculty of Veterinary Medicine, Jordan University of Science and Technology, 22110 Irbid, Jordan; 2 Department of Clinical Medical Veterinary Sciences, Faculty of Veterinary Medicine, Jordan University of Science and Technology, 22110 Irbid, Jordan

**Keywords:** FTA card, RNA preservation, Newcastle disease virus, RNA integrity, ambient temperature

## Abstract

**Introduction:**

The transport of Newcastle disease virus (NDV) specimens, isolates or purified RNA is traditionally performed at ultra-low temperatures using dry ice to prevent degradation. However, this method is costly and requires specialised packaging and stringent shipping conditions. The aim of this study is to evaluate existing products’ capacities to preserve NDV or its RNA under different conditions.

**Material and Methods:**

Flinders Technology Associates (FTA) cards, RNASound cards, and RNAstable tubes were tested for their ability to preserve NDV RNA at ambient temperatures. Two controls – free RNA and free virus – were included for comparison. Preservation was evaluated at various storage conditions (–80°C, –20°C, 4°C, 25°C and 56°C) and incubation times (1, 7, 14, 28 and 35 d) using a reverse-transcription PCR, Sanger sequencing and ratiometric fluorometry.

**Results:**

All preservation methods performed effectively at lower temperatures. The FTA cards maintained consistent RNA integrity with Δ threshold cycles < 2 except at 56°C on days 14–35. RNASound preserved RNA stably but was inconsistent on day 35 at 56°C. RNAstable was effective at intermediate times but had allowed complete degradation by day 35. Free RNA degraded rapidly after day 1, while free virus initially remained stable but deteriorated over time. Sanger sequencing confirmed high-quality recovery, except for recovery of free RNA, which lacked long-term stability.

**Conclusion:**

Despite challenges with prolonged storage and high temperatures, these methods demonstrated satisfactory performance. They offer viable alternatives to ultra-low temperature storage, enabling sample transport at ambient temperatures while preserving RNA integrity, and could be particularly useful in remote settings.

## Introduction

Humans, animals and the environment are key factors in the emergence and spread of various infectious diseases, meaning that interplay between factors is complex, outbreak locations can be multiple and simultaneous, and many early-intervention modes must be weighed up simultaneously, making the rapid analysis of samples to identify causative viruses crucial (20). Molecular assays, such as real-time reverse-transcription (RT)-PCR, provide fast, sensitive and specific detection, and are now widely utilised in diagnostic laboratories to identify a range of viruses, including Newcastle disease virus (25). However, specialised laboratories are often located far from the site of specimen collection, resulting in shipment times that can span several days. Viruses with RNA structure are chemically unstable and vulnerable to degradation by environmental RNases, which can compromise the integrity of the sample. This degradation may hinder a laboratory’s ability to accurately analyse the specimen and provide a reliable diagnosis (10). Virus samples should be stored in virus transport media at low temperatures, ideally frozen at -80°C, for transportation. However, this necessitates the use of dry ice, which has been classified as a “dangerous good” by the International Air Transport Association. As a result, specialised packaging and shipping protocols are required, significantly increasing the cost of transporting samples, particularly from field sites or remote hospitals (19). Thus, there is a growing demand for techniques that can reliably preserve viral RNA at room temperature for extended durations. Such methods would simplify the logistics and significantly lower the costs associated with transporting clinical samples or isolates to diagnostic laboratories.

Several reagents designed to stabilise RNA, such as GenTegra-RNA (GenTegra, Pleasanton, CA, USA) and RNAshell (Imagene, Pessac, France), are effective in maintaining RNA integrity at room temperature. Another such reagent is RNAstable, developed by Biomatrica (San Diego, CA, USA). This product is distinct in its approach, as it is based on the principles of anhydrobiosis, a biological process employed by certain multicellular organisms to survive in a dehydrated state (5, 24). However, these systems require RNA to be pre-extracted, which can be impractical if samples are being sent from a field site or a laboratory with limited technical equipment (24).

Alternative methods for preserving RNA at room temperature have been explored, including the Flinders Technology Associates (FTA) card (GE Healthcare, Rydalmere, NSW, Australia) and the RNASound card (FortiusBio, San Diego, CA, USA). These are specialised filter papers embedded with chemicals that stabilise nucleic acids in clinical samples (4).

The RNASound card offers a simplified RNA extraction and elution process. Virus samples are inactivated upon application to the card’s discs and can be easily eluted by dislodging the discs and shaking them in RNase-free water (13). In contrast, the Whatman paper FTA card is primarily designed for long-term DNA preservation at room temperature, with the manufacturer advising that RNA be processed immediately upon arrival at the laboratory or stored frozen (9). However, RNA from avian influenza viruses can be preserved on FTA cards for up to five months at room temperature (2). Additionally, RNA from certain arboviruses, such as Zika, chikungunya and dengue viruses, can be successfully recovered from FTA cards after 28 days of storage.

In this study, the efficiency of the FTA card, RNAstable and RNASound RNA preservation systems and the degradation of RNA in two controls – free RNA and free virus – was assessed using the LaSota strain of Newcastle disease virus. These preservation techniques and the controls were tested under varying storage temperatures and incubation periods. Following each incubation condition, the preserved RNA was evaluated for integrity and compared to RNA after different incubation conditions based on multiple criteria. The evaluation was made with real-time reverse-transcription (RT)-PCR analysis and Sanger sequencing.

## Material and Methods

### Virus

The LaSota strain of Newcastle disease virus was used as a model virus to evaluate RNA preservation under various storage conditions. The virus was obtained from JOVAC (Amman, Jordan). Both free RNA and free virus samples were utilised as controls to which to compare the effects of different preservation techniques on RNA integrity.

### Placement of virus or RNA in different storage media

**Fig. 1.**
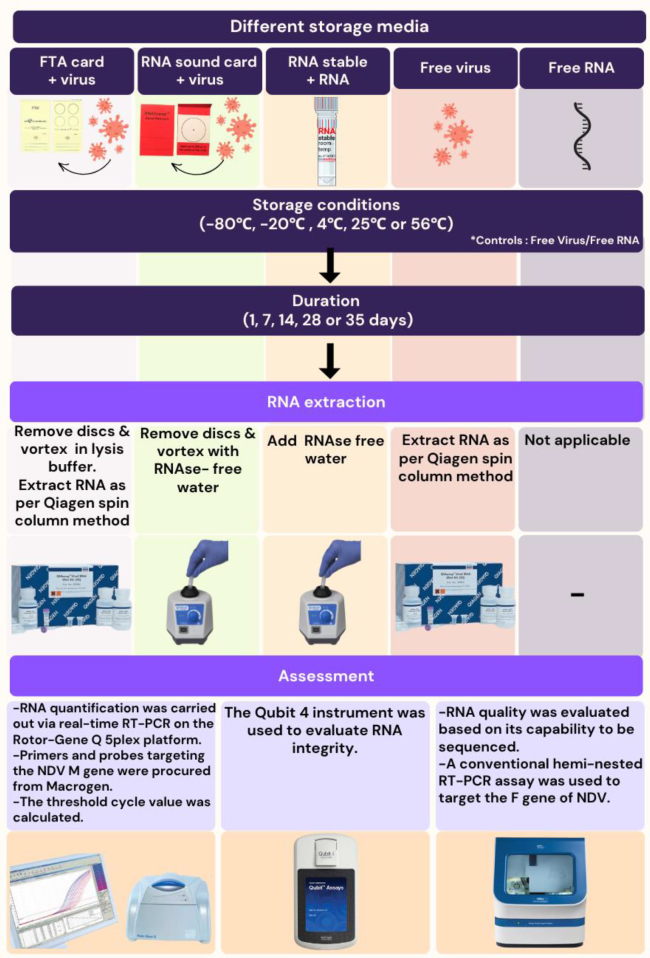
Study methodology. Newcastle disease virus (NDV) RNA preservation using various methods (Flinders Technology Associates (FTA) cards, RNASound cards and RNAstable tubes) across temperatures (–80°C, –20°C, 4°C, 25°C and 56°C) and incubation periods (1, 7, 14, 28 and 35 d). The quality of RNA was assessed using real-time reverse-transcription (RT)-PCR, ratiometric fluorometry and Sanger sequencing

A summary of the different preservation reagents, storage temperatures and time points is presented in Fig 1. Briefly, three RNA preservation products – FTA cards (GE Healthcare, Rydalmere, NSW, Australia), RNAstable tubes (Biomatrica, San Diego, CA, USA) and RNASound cards (FortiusBio, San Diego, CA, USA) – were used for preserving Newcastle disease virus (NDV). Additionally, free RNA and free virus samples served as controls. These methods were tested stored at temperatures of –80°C, –20°C, 4°C, 25°C and 56°C, and through incubation periods of 1, 7, 14, 28 and 35 d. Unless otherwise noted, all RNA purifications and extractions were conducted using the QIAamp Viral RNA Kit (Qiagen, Hilden, Germany). Ten microlitres of NDV virus were added to the FTA and RNASound cards. The same volume but of extracted NDV RNA was added to the RNAstable reagent in tubes. As controls, 25 μL of free NDV virus and 25 μL of RNA were placed into separate 1.5 mL Eppendorf tubes. In total, 125 samples were collected and analysed. To recover the RNA of the virus applied to the FTA card, the disc was placed into a 1.5 mL microtube with type A viral lysis (AVL) buffer and carrier RNA (Qiagen) and vortexed for 5 min, and RNA was extracted according to the manufacturer’s protocol. For recovery from the RNASound card, the push-out disc for each sample was placed into a 1.5 mL microtube with 50 μL of RNAase-free water pre-heated to 75°C, vortexed for 5 min and removed. Ribonucleic acid from free virus was extracted following the kit instructions, while free RNA was used directly.

### Assessment of various NDV RNA preservation methods: real-time RT-PCR

To evaluate RNA quality and titre, real-time RT-PCRs were performed using the Luna Universal Probe One-Step RT-qPCR Kit (New England Biolabs, Ipswich, MA, USA). The reaction mixture for each sample included 10 μL of Luna Universal Probe qPCR Master Mix, 0.8 μL of forward primer (10 μM), 0.8 μL of reverse primer (10 μM), 0.4 μL of probe (10 μM), 5 μL of nuclease-free water and 3 μL of complimentary DNA template. The primers and probe targeted the matrix segment of NDV (11). The thermocycling conditions were as follows: reverse transcription at 55°C for 10 min, initial denaturation at 95°C for 1 min, 45 cycles of denaturation at 95°C for 10 s, and extension at 60°C for 30 s, with plate reading after each cycle. Each sample was run with five standards and one negative control using the Rotor-Gene Q 5Plex thermocycler (Qiagen). The mean threshold cycle (C_t_) value for each sample stored under different conditions was compared to the mean Ct value of the corresponding virus or purified RNA stored at –80°C and expressed as Δ threshold cycle (ΔC_t_). This was calculated as Ct value of the sample – C_t_ value at –80°C at the same time point. Storage at –80°C is considered the gold standard for virus and nucleic acid preservation.

### Assessment of various NDV RNA preservations methods: RNA quality and integrity

To assess the RNA quality and integrity for each preservation method, nine samples were evaluated at different time points (1, 14 and 35 d) and storage temperatures (–80°C, 25°C and 56°C) using the RNA IQ Assay Kit (Cat. No. Q33221; Thermo Fisher Scientific, Eugene, OR, USA) following the manufacturer’s protocol. The RNA integrity and quality (IQ) was measured with a Qubit 4 fluorometer (Life Technologies Holding, Singapore), which provides an RNA IQ score ranging from 1 to 10, with 10 indicating fully intact RNA.

### Assessment of various NDV RNA preservations methods: DNA sequencing of the partial fusion protein gene of NDV

In addition to real-time RT-PCR analysis, RNA preservation was assessed through Sanger sequencing of the partial fusion protein gene (*F* gene) of NDV. For each preservation method, nine samples were selected which were taken at incubation time points of 1, 28 and 35 d and storage temperatures of –80°C, 25°C and 56°C. Conventional RT-PCRs were performed using Platinum Taq DNA polymerase (Invitrogen, Carlsbad, CA, USA). A hemi-nested PCR was carried out with a reaction volume of 25 μL consisting of 2.5 μL of 10× PCR buffer, 1 μL of each forward and reverse primer, 0.1 μL of Taq DNA polymerase, 0.75 μL of MgCl_2_, 0.5 μL of dNTPs, and 18.15 μL of water, along with 1 μL of cDNA template. For the first round of the PCRs, the forward primer MSF1 (5′-GACCGCTGACCACGAGGTTA-3′) and reverse primer NDVR2 (5′-AGTCGGAGGATGTT GGCAGC-3′) were used. In the second round, NDVR2 was combined with an additional forward primer, NDVR7 (5′-TTAGAAAAAACACGGGTAGAA-3′ (3) The thermocycling conditions included an initial denaturation at 94°C for 2 min, 35 cycles of denaturation at 94°C for 45 s, annealing at 50°C for 45 s, extension at 72°C for 1 min and a final extension at 72°C for 7 min. A 500-base-pair PCR product was expected and was confirmed *via* gel electrophoresis.

The PCR products of the partial *F* gene were purified using ExoSAP-IT (Thermo Fisher Scientific Baltics, Vilnius, Lithuania) according to the manufacturer’s protocol. The purified products were sent to Macrogen (Seoul, South Korea) for sequencing, and the resulting DNA sequences were analysed using ChromasPro software (Technelysium, South Brisbane, QLD, Australia).

## Results

### Real-time RT-PCR amplification

**Fig. 2.**
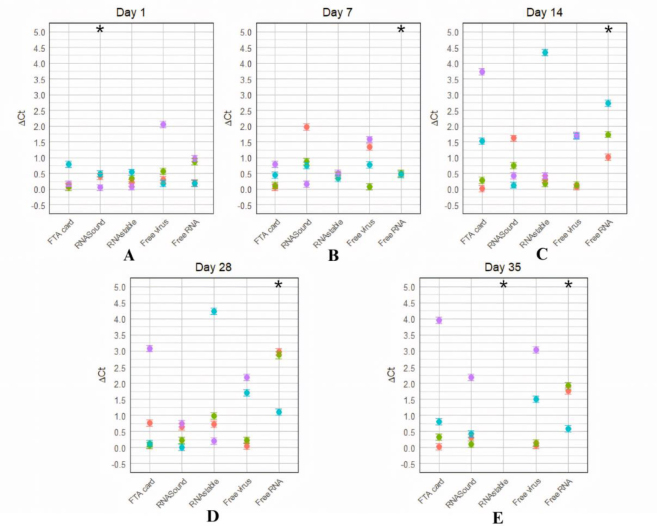
Values of Δ threshold cycle (ΔC_t_) for Newcastle virus RNA samples preserved with Flinders Technology Associates (FTA) cards, RNASound cards and RNAstable tubes or not preserved as free virus and free RNA controls and incubated at –20°C, 4°C, 25°C and 56°C; ΔC_t_s are compared to those of RNA samples stored at –80°C. A – day 1; B – day 7; C – day 14; D – day 28; E – day 35; * – excluded datum or undetectable amplification

The ΔC_t_ values, presented in Fig. 2, assess the effectiveness of each preservation method in maintaining NDV RNA integrity and titre over time.

On day 1, all the NDV RNA preservation methods showed generally similar results across various temperatures, with ΔC_t_ values exhibiting minimal variation. The highest ΔC_t_ observed was approximately 2, indicating minimal RNA degradation and loss in the NDV titre. As an exception, the ΔC_t_ for RNASound at 4°C was significantly elevated because of a technical error, leading to the exclusion of this datum from analysis, as shown in Fig. 2A. These results highlight the importance of early sample collection for accurate real-time PCR data.

Figure 2B shows that on day 7, ΔC_t_ values remained relatively stable across all preservation methods and temperatures, with minimal differences compared to day 1. This consistency indicates effective RNA preservation over the week. However, when no preservation system was in use, significant degradation was observed at 56°C in the free RNA of NDV: the impossibility of obtaining a ΔC_t_ because no amplification could be detected indicates this severe RNA deterioration. Compared to day 1, where ΔC_t_ values were uniformly low, day 7 data reflect a gradual increase in ΔC_t_, illustrating ongoing RNA degradation and emphasising the critical impact of high temperatures on RNA stability.

On day 14 (Fig. 2C) and day 28 (Fig. 2D), ΔC_t_ values varied significantly across FTA cards, RNAstable tubes and the controls and when compared to earlier days, demonstrating the prolonged impact of time on NDV RNA integrity, especially at higher temperatures. Flinders Technology Associates cards and RNAstable tubes exhibited substantial increases in ΔC_t_ values, which reached peaks of 3.73 and 4.34, respectively, in all the data for 25°C and 56°C and days 14 and 28. Newcastle disease virus free RNA continued to show extreme ΔC_t_ values, highlighting severe RNA degradation due to prolonged high-temperature exposure. Free virus and RNASound cards also displayed increasing variability, underscoring the overall decline in RNA preservation effectiveness over time.

By day 35 (Fig. 2E), significant variation in ΔC_t_ values was observed at 56°C, ranging between 2 and a high excluded value, indicating considerable RNA degradation. Notably, RNAstable failed to yield any data in real-time PCR, reflecting severe degradation. Free RNA consistently showed extreme ΔC_t_ values at 56°C, underscoring persistent degradation through prolonged storage. Overall, these results highlight the critical impact of prolonged exposure and high temperatures on RNA integrity across all preservation methods.

Overall, NDV RNA preservation methods demonstrated comparable performance on day 1, except for a technical issue with RNASound cards at 4°C. By day 7, the preservation methods were continuing to perform well, as did the free virus control. On days 14 and 28, significant differences were noted, with FTA cards and RNAstable tubes showing increased ΔC_t_ values, which indicated some degradation but meant that these methods maintained relative efficacy. However, by day 35, RNAstable tubes failed to yield detectable viral RNA, proving the severe impact of prolonged high temperatures on RNA integrity. When the temperature was the highest studied (56°C), exposure of samples to it did not need to be prolonged for RNA to degrade in the case of free RNA; this temperature also generally incubated all samples to the most deleterious extent.

### RNA quality and integrity

The RNA integrity data for preservation methods and controls at three temperatures across three time points (1, 14 and 35 d and storage temperatures –80°C, 25°C and 56°C) are presented in Table 1. Integrity of RNA was quantified on a scale from 1 to 10, where lower values indicate fragmentation of RNA and higher values represent longer, intact RNA.

**Table 1.** Newcastle virus RNA integrity data for various preservation methods and controls at three temperatures across three time points. The integrity of RNA was scored in a range from 1 to 10, where 10 was the highest integrity

	Temperature (°C)	FTA cards	RNASound cards	RNAstable tubes	Free virus	Free RNA
	–80	10	N.D.	9.1	9.4	8.7
Day 1	25	10	N.D.	N.D.	9.0	9.5
	56	10	N.D.	N.D.	8.5	7.9
	–80	10	N.D.	9.1	9.3	8.6
Day 14	25	10	N.D.	N.D.	8.9	9.5
	56	10	N.D.	N.D.	8.3	7.4
	–80	10	N.D.	9.2	8.6	8.6
Day 35	25	10	N.D.	N.D.	8.9	9.4
	56	10	N.D.	N.D.	8.2	7.2

1 FTA – Flinders Technology Associates; N.D. – values not detected

At –80°C across all time points, all preservation methods but RNASound permitted extraction of RNA with IQ above 8.5, indicative of minimal loss of viral RNA integrity. This suggests that –80°C is the optimal temperature for RNA preservation.

Flinders Technology Associates cards consistently demonstrated the highest RNA IQ among all methods at every time point and temperature. In contrast, RNAstable only provided a satisfactory RNA IQ at –80°C, and the RNA IQ from this method was significantly compromised at the temperatures of 25°C and 56°C. For the controls, both Free virus and Free RNA started with higher integrity scores at low temperatures, but their RNA IQ values declined at 56°C, falling to 8.2 and 7.2, respectively.

### DNA sequencing of the partial fusion gene of NDV

To evaluate the preservation methods, samples from different preservation methods and controls (1, 14 and 35 d and storage temperatures of –80°C, 25°C and 56°C) were sent for sequencing, and the results were compared to a reference sequence to determine if the RNA was preserved. The data are presented in Table 2.

**Table 2.** Reference sequence to partial F gene sequence match results after Newcastle virus RNA preservation as free virus and free RNA and by Flinders Technology Associates (FTA) card, RNASound and RNAstable

	Free virus		FTA cards		RNASound cards		Free RNA		RNAstable tubes	
	Sample number	Treatment (°C)/days	Sample number	Treatment (°C)/days	Sample number	Treatment (°C)/days	Sample number	Treatment (°C)/days	Sample number	Treatment (°C)/days
	1	–80/1	10	–80/1	19	–80/1	28	–80/1	37	–80/1
	7	–80/35	11	25/1	20	25/1	29	25/1	38	25/1
	8	25/35	12	56/1	21	56/1	30	56/1	39	56/1
	-	-	14	25/28	22	–80/28	31	–80/28	40	–80/28
	-	-	15	56/28	24	56/28	32	25/28	41	25/28
	-	-	16	–80/35	26	25/35	34	–80/35	42	56/28
	-	-	17	25/35	27	56/35	35	25/35	43	–80/35
	-	-	-	-	-	-	-	-	44	25/35
	-	-	-	-	-	-	-	-	45	56/35
Matching samples/total samples	-	3/9	-	7/9	-	7/9	-	7/9	-	9/9

At the three selected time points and the three temperatures, positive results were obtained universally, including from the controls. RNAstable showed the best performance, with all 9 out of 9 samples being viable for sequencing and giving the expected results for the partial *F* gene sequence, making it the most reliable for sequencing. The FTA card and RNASound card methods and Free RNA each had 7 out of 9 with good sequencing results. Free virus had the poorest outcome, with only 3 out of 9 permitting partial *F* gene sequences to be generated, indicating significant degradation, particularly at 25°C and 56°C over extended times.

## Discussion

Preserving viral RNA during transportation is especially challenging when samples are collected from remote locations with limited access to specialised laboratories. Current methods require freezing samples at –80°C, which involves the use of dry ice, classified as a dangerous good. This results in high costs and complex logistics for shipping. The issue is particularly critical for RNA viruses, such as the enveloped Newcastle disease virus, which are vulnerable to degradation during transit times. In this study, we aimed to evaluate the effectiveness of the FTA card, RNASound card and RNAstable tube viral RNA preservation methods, with two controls (free RNA and free virus) for comparison, focusing on their ability to preserve Newcastle disease virus LaSota strain RNA. These preservation methods were tested under different storage temperatures and time intervals to simulate real-world conditions. Following each storage period, we assessed NDV RNA quality, integrity and titre through real-time RT-PCR analysis, fluorometric measurement and DNA sequencing of the partial fusion gene to determine the overall efficiency and reliability of each method.

Overall, all preservation methods for NDV virus or RNA and the controls demonstrated good performance at lower temperatures. However, differences in their preservation performance became more apparent with prolonged incubation periods and increased temperatures, as revealed through the real-time RT-PCR, fusion gene sequencing and fluorometric RNA integrity measurement, which provided criteria to assess RNA quality and which have been employed in similar studies (13, 24).

In the real-time RT-PCR analysis, we measured the differences in C_t_ values for each preservation method, and for the control samples (free virus and free RNA) across various time points and incubation temperatures and compared them to the C_t_ values of samples stored at –80°C at each time point. The paper-based preservation methods (FTA cards and RNASound cards) and the RNAstable liquid solution demonstrated excellent preservation performance during the first week of incubation, yielding ΔC_t_ values of approximately 2. The FTA card gave a smaller ΔC_t_ for all time points and all tested temperatures except at 56°C on days 14, 28 and 35, when the ΔC_t_ ranged from 3 to 4. The other paper-based preservation method (RNASound) was more consistent at all tested time points and temperatures except on day 35 at 56°C, when the ΔC_t_ was around 2. The liquid-based preservation method (RNAstable) showed interesting results, with great performance at the 14- and 28-day points (except at 25°C) and then total disappearance of the NDV RNA at all tested temperatures on day 35. Free NDV virus had a smaller ΔC_t_ on days 1 and 7 at all tested temperatures, while on days 14, 28 and 35, the ΔC_t_ was smaller for the lower temperatures of –20°C and 4°C. By day 35, both the FTA card and RNAsound experienced a decline in RNA quality, with ΔC_t_ values reaching nearly 4. Notably, RNASound exhibited a significantly elevated ΔC_t_ value even on the first day of the 4°C sample collection, likely because of technical errors during sample handling or poor storage conditions. Overall, FTA cards and RNASound performed well across all temperatures, which aligns with existing literature on advanced nucleic acid preservation techniques. For instance, RNA from the avian influenza virus can be stored at room temperature using FTA cards for up to five months (13), and RNA from the Zika, chikungunya and dengue arboviruses can be successfully recovered from FTA cards even after 28 days (8). Similarly, the RNASound card effectively preserved influenza virus RNA for approximately 28 days at room temperature (13). Other preservation methods, such as those using spin column techniques, have also kept RNA stable at room temperature, as evidenced by MERS-CoV studies. In these studies, the average starting C_t_ value at week 0 was 18.524 ± 0.84, increasing to 20.25 ± 1.82 at week 16, indicating a difference of approximately 1.73 between the baseline C_t_ and the value recorded 16 weeks later (1). When used in our experiment, both of these methods exhibited comparable ΔC_t_ values.

RNAstable demonstrated promising results in real-time PCR analyses; however, by day 35, it exhibited extreme ΔC_t_ values across all storage conditions, highlighting a significant drawback of this technique. This issue mirrors findings by Stevens *et al*. (23), who encountered similar challenges while stabilising HIV viral RNA. They noted that an incipient in the product formulation adversely affected the real-time PCR analysis, possibly because it quenched the fluorescent signal.

The controls employed in the real-time PCR evaluation performed as anticipated. The free RNA remained intact only on day 1, after which it completely degraded and did not yield any C_t_ values. This may be ascribed to the inherent instability of RNA compared to DNA and the presence of RNA-degrading enzymes (RNases) (10). In contrast, the free LaSota strain NDV exhibited low ΔC_t_ values, comparable to those of the preservation methods tested. This observation underscores the significance of nucleic acid stability, as lyophilisation is commonly utilised in vaccine production to preserve viral antigens and adjuvants, thereby extending their shelf life (18).

Degradation of RNA occurs gradually, making the evaluation of RNA integrity crucial for obtaining reliable gene expression data. Agilent Technologies has introduced a novel RNA quality assessment tool, the RNA integrity number (RIN), which standardises RNA quality control through a numeric scale of 1 to 10, where 1 indicates maximum degradation and 10 signifies complete integrity (17). In our study, we utilised RNA integrity and quality assessment, which is reported similarly to the RIN. The IQ method employs two unique dyes: one that specifically binds to large and/or highly structured RNA, and another that targets small, degraded RNA (14). Measurements were taken using the Qubit 4 machine with the RNA IQ assay kit under conditions and at time points consistent with those adopted for RT-qPCR. The RNA from the FTA cards scored perfect 10s for integrity across all conditions and time points, demonstrating the cards’ capacity to preserve RNA integrity. Although limited research has evaluated the RIN for FTA cards, some studies report successful PCR results in all samples extracted from FTA Classic and Elute cards (22). Additionally, another study noted a relationship between RNA stability and ΔC_t_ values, with gradual degradation observed over 30 days at 37°C, while no significant differences in RNA amounts were found for samples stored at 4°C and 25°C (12). Similarly, lymph node fine needle aspiration samples stored for up to 123 days produced both 500-base-pair and 1.5-kilobase-pair amplicons in PCRs with nucleic acid extracted from FTA cards, indicating high nucleic acid integrity (22). Also, Manswr *et al*. (15) and Moscoso *et al*. (16) demonstrated the preservation of infectious bronchitis virus RNA for 21 and 36 days, respectively, at room temperature using FTA cards. Collectively, these studies suggest that FTA cards effectively maintain RNA integrity over extended periods and varying temperatures.

Although RNA samples provided reliable results during RT-qPCR, they did not exhibit a detectable RIN, which may be attributed to technical errors, improper sample handling, inadequate storage conditions or issues with kit preparation. In contrast, RNAstable at –80°C maintained a RIN of 9.2 across all time points. However, at elevated temperatures, the RIN became undetectable. A related study evaluated RNA stability in RNAstable after 4.5 months and 11 months of storage at room temperature and 45°C compared to a control stored at –80°C. The results indicated high RIN values of 9.8 for the stabilised RNA over the shorter storage period and 9.5 for the control. While the RIN of the stabilised RNA decreased from 9.8 at 4.5 months to 8.6 at 11 months, it remained within the range of excellent integrity (24).

The controls in the present experiment behaved as anticipated, beginning with high RIN values above 7.8 on day 1 across all temperatures. Although there was a slight decline in RNA quality by day 35, values remained above 7.1. Notably, at the low temperature of –80°C, the RIN averaged nearly 9 for both samples. While temperature did impact RNA integrity, there was still some retention of intact RNA.

Sanger DNA sequencing has long been regarded as the gold standard for determining nucleic acid (NA) sequences, whether they are naturally occurring or synthetically produced (6). This method not only serves as a confirmatory assay with high diagnostic specificity but is also crucial for the initial development of molecular assays. High-quality NA sequence analysis is essential for monitoring the efficacy of these assays and their components, as well as for evaluating the preservation methods used to ensure clear sequencing results. It is important to recognise that the quality of the PCR assay used to generate the sequencing target – encompassing primer design and the optimisation of reagents and conditions – significantly influences the quality and quantity of the resulting DNA or complimentary DNA amplicons for sequencing analysis (21). In our study, the three methods produced optimal results much of the time, with at least seven out of nine samples matching the reference, demonstrating consistent outcomes across various temperatures and time points. This suggests that PCR and sequencing reliably assess the integrity of the preserved virus/RNA methods. However, one control, free RNA, exhibited similar preservation characteristics, while the free virus control was negatively impacted by high temperatures and extended storage times, yielding only three reference-matching samples out of nine. This highlights the importance of storage duration and temperature, fixatives in the substrate and other treatments (such as demineralisation) on the stability and quality of RNA when it is preserved in diagnostic materials (6, 7).

## Conclusion

Despite some drawbacks associated with extended storage periods, higher temperatures and potential technical errors during the extraction process, all three commercial RNA preservation methods demonstrated satisfactory performance and met quality assessments. These methods offer a viable alternative to the challenging transportation of samples at ultra-low temperature, particularly in remote locations. They enable the shipping of samples at ambient temperatures while maintaining RNA integrity, facilitating a range of molecular assays.
